# Interactions Between the Prefrontal Cortex and Attentional Systems During Volitional Affective Regulation: An Effective Connectivity Reappraisal Study

**DOI:** 10.1007/s10548-015-0454-2

**Published:** 2015-10-06

**Authors:** Tomasz S. Ligeza, Miroslaw Wyczesany, Agnieszka D. Tymorek, Maciej Kamiński

**Affiliations:** Psychophysiology Laboratory, Institute of Psychology, Jagiellonian University, Ingardena 6, 30060 Kraków, Poland; Institute of Zoology, Jagiellonian University, Gronostajowa 9, 30387 Kraków, Poland; Department of Biomedical Physics, Faculty of Physics, University of Warsaw, Pasteura 5, 02093 Warsaw, Poland

**Keywords:** Emotional regulation, Reappraisal, Prefrontal cortex, Connectivity, Attention

## Abstract

Reappraisal is an emotion regulation strategy used to change reactions to emotion-related stimuli by reinterpreting their meaning. During down-regulation of negative emotions, wide areas of the prefrontal cortex (PFC) inhibit emotion-related brain areas such as the amygdala. Little is known, however, about how this control activity influences the earliest stages of affective responses by modulating perceptual and attentional areas. The aim of this study is to identify the connectivity patterns between the PFC and the core regions of two well-known attentional networks: the dorsal attentional network (which controls attention volitionally) and the ventral attentional network (which controls attention spontaneously) during reappraisal. We used a novel method to study emotional control processes: the directed transfer function, an autoregressive effective connectivity method based on Granger causality. It was applied to EEG recordings to quantify the direction and intensity of information flow during passively watching (control condition) or reappraising (experimental condition) negative film clips. Reappraisal was mostly associated with increased top-down influences from the right dorsolateral PFC over attentional and perceptual areas, reaching areas including dorsal attentional regions. The left dorsolateral PFC was associated with the activation of the ventral attentional network. Passively watching clips (control condition) resulted in increased flow from attentional areas to the left dorsolateral PFC, what is interpreted as a monitoring process. Thus, reappraisal seems to be related to both volitional and automatic control of attention, triggered by the right and left dorsolateral PFC respectively.

## Introduction

The study examines the involvement of the prefrontal cortex (PFC) in the process of emotional control by assessing the EEG effective connectivity. As the method makes it possible to determine the direction and causality of influences between cortical regions, it is expected to reveal the ways in which top-down control is involved during the condition of volition emotional modulation. In particular, we examine how it modulates the perceptual and attentional systems.

One of the best studied volitional form of emotional control is reappraisal. It is a cognitive strategy used to change the affective response to emotionally-laden stimuli by reinterpreting their meaning (Gross and John 2003). For example, when viewing a film clip of a car accident, one can downregulate an upcoming emotional response by taking a third-person perspective (e.g. “I am documenting this event as a photographer”) or imaging a positive outcome (e.g. “All accident victims will recover fully soon”). Therefore, the goals of reappraisal are similar to those of cognitive behavioral therapy. Successful reappraisal decreases measures of negative emotions, both experiential and physiological, and is associated with adaptive long-term improvement in everyday functioning (Dillon and Labar 2005). In most fMRI/PET studies, implementation of reappraisal activates wide areas of the PFC, including dorsolateral, ventrolateral, dorsomedial and ventromedial parts (DLPFC, VLPFC, DMPFC, VMPFC) (Buhle et al. 2013; Ochsner and Gross 2005).

Prefrontal activations associated with reappraisal control processes regularly show negative correlations with the emotion-related brain areas, such as the amygdala or the insula; it is proposed that the inhibition signals originate in the prefrontal control centres (Cho et al. 2012; Delgado et al. 2008). However, little is known about how these control regions affect other cortical areas, especially related to the early stages of affective perceptual processing. It is proposed that the attentional gating mechanism plays an important role in the overall modulation of affective responsiveness (Wyczesany et al. 2014a, b).

Thus, to fully understand the early phases of affective modulation, one should consider the complex nature of the attentional network. It comprises two distinct systems. The dorsal attentional network (DAN) maintains the locus of attention relevant to the current task or goal-related requirements, so it is expected to be primarily associated with volitional efforts. Anatomically, it includes bilateral frontal eye fields (FEF) and intra-parietal sulci with superior parietal lobules (IPS/SPL) (Corbetta and Shulman 2002). This network can exert specific top-down control over the perceptual system, from primary to association visual areas (Ruff et al. 2008). The ventral attentional network (VAN) responds when initially unattended or unexpected objects of behavioral relevance are detected, so it is related to the bottom-up, stimuli-driven influences. Core regions of this network are mainly located in the right hemisphere: part of the middle and inferior frontal gyrus (MFG/IFG), anterior insula (aIns), and temporoparietal junction (TPJ). This network may regulate attention spontaneously, in absence of any voluntary efforts (Viviani 2013).

The aim of this study is to identify patterns of effective connectivity related to the reappraisal of negative stimuli and to explore how volitional control of emotions modulates the earliest stages of affective response originating in perceptual and attentional systems. We apply conditions of either passive attending or reappraising (downregulating) emotions evoked by film clips. The passive attention condition serves as control to assess the patterns of effective connectivity related to the processing of negative stimuli without controlling them. To our knowledge, no other study has used the EEG connectivity method to estimate the effects of reappraisal. So far, the majority of similar studies have used fMRI as a measure; however, this method remains dubious for drawing conclusions on the causality of brain activations. Even more advanced fMRI effective connectivity techniques are susceptible to the variation and lag of hemodynamic responses and, as a result, their usefulness is still under discussion (Smith 2012). In contrast, EEG recordings, which directly reflect neural activity, make it possible to draw more direct conclusions on effective connectivity and network interactions. Moreover, EEG provides far more natural measurement conditions, which are crucial when exploring emotional processes. In the present study, the directed transfer function (DTF) was used to conduct an effective connectivity analysis. This method estimates the direction and strength of information flow between selected brain regions, allowing us to draw conclusions on their causal relationships (Kamiński and Blinowska 1991).

While the current understanding of reappraisal is mainly based on reinterpreting emotional pictures, this study uses more ecologically valid film clips, which had been used in few experiments (Goldin et al. 2008). We also include self-report measurements of the subjective affect to control the experimental effect as well as individual differences in the efficiency of emotional control and their potential influence on connectivity patterns.

We hypothesize that reappraisal compared to no-control negative conditions is mainly associated with increased influences originating in the prefrontal regions towards perceptual and attentional areas. These influences are expected to activate the dorsal and ventral attentional networks which represent volitional and more automatic control of attention. The passive watching condition is expected to trigger flows related to emotional or monitoring processing with bottom-up influences.

## Materials and Methods

### Procedure

The procedure was compliant with the directives of the Helsinki Declaration (1975, revised 2000) and approved by the Ethical Committee of the Institute of Psychology, Jagiellonian University. All participants were warned that the film clips may present strongly affective content. 33 women volunteers (median age: 21, range 18–33) participated in the study after signing a written consent form. All volunteers were medication-free with no reported history of any neurological or psychiatric disorders or substance abuse, and had normal or corrected-to-normal vision. During the procedure, the subjects were seated in an air-conditioned soundproof cabin in front of a 24″ LCD monitor. We used the Biosemi ActiveTwo system with 64 electrodes placed on a 10–10 headcap, with four additional leads for monitoring eye movement and two more electrodes for offline linked mastoid reference.

During a prior experimental session, the participants received in-depth training in reappraisal while viewing standardized emotional pictures. They were taught how to generate more positive interpretations of the scenes in three ways: (1) by denying their reality, (2) by taking a third-person perspective, or (3) by imaging their positive outcomes. Participants were free to use any of these strategies to control their emotions in the most efficient way. During the training session, they were instructed to use reinterpretation through several trials, with feedback and shaping by the experimenter (during some trials, the subjects were asked to report their reinterpretations aloud). When a cue word (“control” or “watch”) appeared on the screen, they were taught to reappraise or to simply view the picture and respond naturally, letting their emotions arise. The training session lasted until the participants declared they are ready to proceed with the experiment based on the film clips. The subjects were also asked to implement the strategy only after the picture/film appeared on the screen and to fully watch its content, without closing their eyes or looking away.

Thirty-six 20 s-long film clips were chosen, aiming to feature balanced content, including 12 neutral (normal food, people in everyday activities or ordinary relationships, animals, beauty treatments, neutral landscapes) and 24 negative clips (disgusting food, people in dramatic situations, fights, animal slaughter, mutilations, surgical procedures, accidents). The clips were presented randomly in three runs of fixed-ordered blocks (conditions): NEU (watching neutral clips), NEG (passively watching negative clips), and REAP (reappraising negative clips). Each block consisted of four clips. During the clips, an additional hint was displayed to remind the participants about the current experimental conditions. The block design was chosen to minimize extra brain activations associated with the effort of switching between the tasks. At the end of each block, the subjects were asked to assess their current affective experience regarding valence, intensity and dominance associated with their emotions using a computerized version of the Self Assessment Manikin. Additionally, after the main procedure, the subjects were asked to estimate the negativity of each film clip and to report any failures in their reinterpretation of particular clips.

### Data Analysis

We assumed that the manifestations of voluntary emotional control will be visible in the contrast of “reappraisal” minus “passively watching negative clips” conditions (these conditions differ only in terms of the instruction received: to reappraise or to watch the clips naturally), while the manifestation of processing or monitoring of emotionally-loaded content will be visible in the contrast of “passively watching negative clips” minus “watching neutral clips” conditions. Since our main connectivity analyses were related to a few directional hypotheses we used planned t-contrasts for ultimate verification of the expected differences between conditions for both behavioral and connectivity data.

### Behavioral Analysis

Two *t* tests reflecting two planned contrasts were used to determine the effect of each condition on the valence of self-reported affect.

As the ability to regulate negative emotions depends on individual and situational characteristics, in order to control this variability, we calculated an additional variable: *effectiveness of control*. It was estimated based on the valence ratings from the Self Assessment Manikin test. For each participant we calculated the difference between mean valence ratings after “reappraisal” blocks and mean valence ratings after “passively watching negative clips” blocks. Arousal and dominance ratings were not included in the effectiveness of the control variable, as emotional control is mainly targeted towards the valence dimension. Thus, the *effectiveness of control* variable reflected the within-group variation in participant reappraising ability, motivation, and involvement in the procedure. This variable was used as a covariate in further analysis.

### Connectivity Analysis

The DTF was used to conduct the connectivity analysis. The DTF method is based on the multivariate autoregressive model (MVAR). In this approach we assume that a sample of the data in *k* channels at a time *t* can be expressed as a weighted sum of *p* previous samples with a random component added:

1 $$X(t) = \sum\limits_{j = 1}^{p} {A(j)X(t - j) + E(t)}$$

where $X(t) = (X_{1} (t),X_{2} (t), \ldots ,X_{k} (t))^{T}$ is the vector of data values at the time *t* and $E(t) = (E_{1} (t),E_{2} (t), \ldots ,E_{k} (t))^{T}$ is the vector of the random component values at the time *t*. The matrices **A**(*j*) (of size *k* × *k*) are known as the model parameters (or coefficients) and *p* is known as the model order.

This approach is well established in EEG data analysis and has many advantages. Most importantly, it makes it possible to construct a measure of causal relations between signals *X*_*i*_(*t*) based on the Granger causality concept (Granger 1969). Granger causality defines a signal *Y* as causal for a signal *X* if values of *X* can be better predicted using previous values of both signals *X* and *Y* than using previous values of signal *X* alone. In particular, DTF estimates derived from MVAR parameters can be interpreted as causal relations. Another advantage of the MVAR approach is that it accounts for the whole multivariate set of signals, so the analysis is not performed separately for every pair of signals (pair-wise). This way we avoid problems which may arise in the event of a presence of common sources in the set of signals, which may lead to a misinterpretation of results (Kus et al. 2004) Moreover, because volume conduction propagates nearly instantly, it does not involve a time delay between signals and does not increase transmission in DTF results (Kamiński and Blinowska 2014). This greatly improves spatial resolution as compared to a typical EEG analysis, which has been confirmed by several neuroimaging studies in different domains (Brzezicka et al. 2010; Kamiński and Blinowska 2014).

The MVAR model can be transformed to the frequency domain where it takes a form of a linear filter **H** with noise **E** on input and signal **X** on its output:

2 $$\begin{array}{*{20}c} {X(f) = A^{ - 1} (f)E(f) = H(f)E(f)} \\ {H(f) = \left( {\mathop \sum \limits_{m = 0}^{p} A(m)\exp ( - 2{{\pi {\rm imf}\Delta }}t)} \right)^{ - 1} } \end{array}$$

where **X**(*f*), **A**(*f*) and **E**(*f*) are the Fourier transform of **X**(*t*), **A**(*j*) and **E**(*t*), respectively. The matrix **H**(*f*) = **A**^−1^(*f*) is known as the transfer matrix. Details of the procedure can be found in Kamiński and Blinowska (1991).

The normalized DTF function is defined in the frequency domain as:

3 $$\gamma_{\text{ij}}^{2} (f) = \frac{{\left| {H_{\text{ij}} (f)} \right|^{2} }}{{\mathop \sum \nolimits_{m = 1}^{k} \left| {H_{\text{im}} (f)} \right|^{2} }}$$

where *H*_*ij*_(*f*) are elements of the transfer matrix **H**. Normalized DTF γ_*ij*_(*f*) describes the causal influence of channel *j* on channel *i* at frequency *f*. The above equation defines a normalized version of DTF, which takes values from 0 to 1 producing a ratio between the inflow from channel *j* to channel *i* to all the inflows to channel *i*.

The preprocessing of the EEG signal was performed using the EEGLab toolbox which included downsampling to 128 Hz. Next, beta band frequencies were extracted using zero-phase 13 Hz high-pass and 30 Hz low-pass filters. The beta band has been chosen as the flows can be characterized as moderate or long distance cortical connections. It has been proposed that such connections are based, above all, on beta oscillations (Bastos et al. 2012; Vossel et al. 2013). This is also consistent with the evidence of the crucial role of beta oscillations in attentional processes, especially in the visual domain (Gola et al. 2013; Kamiński et al. 2012; Kus et al. 2008; Wróbel 2000). Additionally, our previous studies based on the DTF measure also confirmed the importance of the beta band in cortical communication (Wyczesany et al. 2014a, b).

Since the signal subjected to the DTF analysis should not be modified by any method which alters the original correlation structure of the dataset, we did not apply any artifact correction method. Instead, the fragments of signals contaminated with artifacts were rejected. To obtain this, the 20 s-long recordings from each movie clip were divided into 2 s-long epochs which were screened for artifacts. First, the independent component analysis (ICA) was used to separate the eye movement signal, identified using criteria of scalp distribution and temporal characteristics (Jung et al. 2000). Trials in which the amplitude of eye components exceeded 30 μV were rejected. Second, trials where the amplitude on any electrode exceeded 100 μV were also rejected (average 26.6 per subject, mostly due to eye-blinks). Moreover, the epochs for which the subjects reported failures in reinterpretation were also excluded (average 1.33 film clip per subject). Due to statistical constraints, we reduced the set of simultaneously processed signals to 24 channels (provided below). 256-sample long (2 s), artifact-free epochs were analyzed. Then, in order to ensure that the remaining data length after all above processing steps was sufficient to proceed with MVAR modelling, the following formula was applied: W ≥ 10 * pM/N (where W is the required minimum window size in samples, p is the model order, M is the number of channels and N is the total number of epochs; Korzeniewska et al. 2003). DTF calculations were carried out using Multar software (University of Warsaw). The MVAR model order was set to six, according to the Akaike criterion (AIC). Normalized DTF values were then determined. In order to compare propagations for the two experimental conditions, the DTF results were integrated over the selected beta frequency band (13–30 Hz). The distributions of the DTF values were checked to identify and reject possible extremes, defined using boxplot 1.5 IQR (interquartile range). Based on brain atlas (Kaiser 2007; Okamoto et al. 2004), the following regions of interest (ROIs) were defined: anterior orbitofrontal cortex (OFC: AF7, AF8, Fpz); left/right dorsolateral PFC (LDL: F3, F5; RDL: F4, F6); anterior cingulate (ACC: FCz, Fz); right inferior frontal gyrus (RIFG: F8, AF8); left/right intraparietal sulcus (LIPS: CP1, P3, P1; RIPS: CP2, P4, P2); right temporoparietal region (RTPJ P6, P8, TP8); and occipital area (Occ: O1, O2, PO3, PO4). In spite of some concerns regarding the quality of EEG recording from deeper structures like the anterior cingulate, we decided to consider this region in our analyses, due to its importance in emotional control and affective phenomena. It was further justified by the fact, that in the classic EEG analysis techniques, the ACC activity is widely analyzed (just to mention the ERN or N2b components of evoked potentials; Herrmann et al. 2004).

To estimate the effect of condition on information flow between two given ROIs, a one-way repeated-measures MANCOVA analysis was run. It included all combinations of flows between all electrodes in the source and the destination ROI as dependent variables, and the condition as an independent factor. Furthermore, in order to minimize the impact of individual differences on the performance of emotional control, another analysis was run with *effectiveness of control* as a covariate. This covariate was used only when exploring effects related to reappraising. Additionally, the tests were followed by planned comparisons reflecting the hypothesized directionality of flows.

## Results

### Behavioral Data (Manipulation Check)

The mean valence after “watching neutral clips” blocks (M = 0.71, SD = 1.56) was significantly higher i.e. more positive (t(32) = 10.03,* p* < 0.001) than for “passively watching negative clips” blocks (M = 0.39, SD = 1.78). The mean valence of emotions experienced after “reappraisal” blocks (M = 0.44, SD = 1.71) was significantly higher i.e. more positive (t(32) = 2.30, *p* = 0.028) than the mean valence after “passively watching negative clips” blocks (M = 0.39, SD = 1.78). No significant effects of condition for mean arousal and dominance were found.

### Connectivity Data

**Fig. 1:**
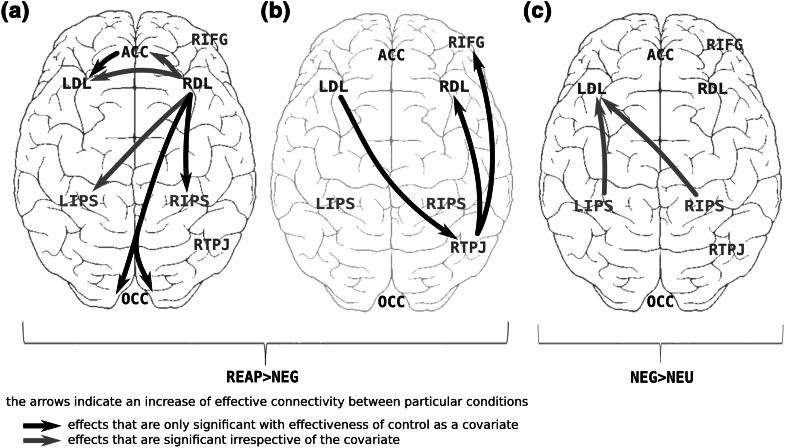
DTF flows were significantly increased between the selected conditions: REAP versus NEG and NEG versus NEU

In comparison to the “watching neutral clips” condition, the “passively watching negative clips” condition (processing or monitoring of emotionally loaded content, covariate excluded) elicited significantly stronger flows from the left and right intraparietal sulci (RIPS, LIPS) to the left dorsolateral PFC (DLPFC) (mean DTF value for NEG condition = 20.81 and 8.57, respectively, and for NEU condition = 18.51 and 5.99, respectively; planned comparison significance: *p* = 0.009; *p* < 0.001, respectively). In comparison to the “passively watching negative clips” condition, the “reappraising negative clips” condition (volitional control of emotions, covariate excluded) revealed significantly stronger flows in the following directions: from the right dorsolateral PFC (DLPFC) to the left intraparietal sulcus (LIPS) (mean DTF for REAP = 2.92; mean DTF for NEG = 1.90) (*p* = 0.01), to the anterior cingulate cortex (ACC) (mean DTF for REAP = 8.67; mean DTF for NEG = 5.71) (*p* = 0.009) and to the left DLPFC (mean DTF for REAP = 5.28; mean DTF for NEG = 3.62) (*p* = 0.016). With the effectiveness of control taken as a covariate, further effects were significant: from the right DLPFC to the right IPS (mean DTF for REAP = 7.61; mean DTF for NEG = 4.48) (*p* = 0.024) and to the occipital cortex (OCC) (mean DTF for REAP = 2.72; mean DTF for NEG = 1.76) (*p* = 0.017); from the left DLPFC to the right temporoparietal junction area (RTPJ) (mean DTF for REAP = 2.15; mean DTF for NEG = 1.16) (*p* = 0.025); from the RTPJ to the right inferior frontal gyrus (RIFG) (mean DTF for REAP = 12.32; mean DTF for NEG = 8.56) (*p* = 0.018) and to the right DLPFC (mean DTF for REAP = 4.21; mean DTF for NEG = 3.14) (*p* = 0.055); and from the ACC to the left LDL (mean DTF for REAP = 18.29; mean DTF for NEG = 14.42) (*p* = 0.036). The significant effects of conditions on the intensity of information flow measured by DTF are depicted in Table 1 and Fig. 1.

**Table 1:** DTF values and statistics

Flow direction	DTF mean (SE)		Omnibus test			Planned comparisons REAP minus NEG
	REAP condition	NEG condition	df	F	p	p
LDL → RTPJ^a^	2.15 (0.52)	1.16 (0.19)	2.25	4.88	0.014	0.025
RDL → LDL	5.28 (0.84)	3.62 (0.50)	2.25	3.17	0.054	0.016
RDL → RIPS^a^	7.61 (1.80)	4.48 (0.93)	2.25	3.12	0.056	0.024
RDL → LIPS	2.92 (0.57)	1.90 (0.34)	2.25	4.53	0.018	0.010
RDL → OCC^a^	2.72 (0.42)	1.76 (0.26)	2.25	4.01	0.031	0.017
RDL → ACC	8.67 (1.38)	5.71 (1.00)	2.34	5.15	0.011	0.009
ACC → LDL^a^	18.29 (3.68)	14.42 (2.94)	2.36	3.22	0.052	0.036
RTPJ → RDL^a^	4.21 (1.06)	3.14 (0.85)	2.28	5.87	0.007	0.018
RTPJ → RIFG^a^	12.32 (2.54)	8.51 (1.31)	2.44	3.15	0.053	0.055

Flow direction	DTF mean (SE)		Omnibus test			Planned comparisons NEG minus NEU
	NEG mean (SE)	NEU mean (SE)	df	F	*p*	*p*
LIPS → LDL	20.81 (2.60)	18.51 (3.084)	2.33	5.03	0.011	0.009
RIPS → LDL	8.57 (1.09)	5.99 (0.721)	2.28	5.55	0.009	<0.001

Both the omnibus test and planned comparisons are provided

*REAP* reappraisal condition, *NEG* watching negative condition, *NEU* watching neutral condition

^a^The tests which were only significant with reappraisal efficiency included as a covariate

## Discussion

This EEG study investigated the role of the PFC in the affective control phenomenon by using effective connectivity analysis. We examined the causality and direction of communication between selected cortical regions during downregulating (reappraisal) or passively watching emotionally arousing film clips. As expected, reappraising was associated with top-down influences from the right DLPFC on the attentional and perceptual regions, reaching, among others, the dorsal attentional network. Moreover, during reappraisal, the left DLPFC was associated with the activation of the ventral attentional network. Interestingly, most of the reappraisal effects were visible only when the efficiency of control was considered. Passive watching conditions triggered putative monitoring flows from the dorsal network to the left PFC.

The most prominent effect was associated with increased outflow from the right DLPFC during volitional control of negative film clips (“reappraisal” condition) compared to conditions with no explicit control instruction (“passively watching negative clips” condition). The outflow targeted a number of cortical regions: occipital cortex (OCC), bilateral intraparietal sulcus (LIPS, RIPS), anterior cingulate area (ACC), and left dorsolateral cortex (LDL) (Fig. 1, graph “a”). The increase in signal propagation to the occipital and parietal regions may indicate a heightened top-down control enforced by the right DLPFC on perceptual and attentional systems. This may reflect an involvement of the sensory gating mechanism, which provides modulation of the initial stages of emotional information processing. Such early influences are considered to be a primary modulatory mechanism regulating emotional responsiveness (Wyczesany et al. 2015). Other data further support this reasoning as the direct epidural stimulation of the DLPFC decreases the late positive potential (LPP), the marker of visual attention to emotionally salient stimuli (Hajcak et al. 2010). Thus, as expected, reappraisal seems to affect the dorsal attentional network (DAN) which maintains the goal-oriented locus of attention by exercising top-down control via the DLPFC (Corbetta and Shulman 2002). Anatomical data shows that the DLPFC is unlikely to exert a strong direct influence on amygdala processing (Ray and Zald 2012). Thus, increased flow to the ACC may represent indirect influences on subcortical emotion regions, as the ACC region has the largest output to the amygdala of the PFC regions (Ghashghaei et al. 2007).

Reappraisal was also related to the increased information flow from the left DLPFC to the right temporoparietal junction area (RTPJ), and further from the RTPJ to the inferior frontal gyrus (IFG) (Fig. 1, graph “b”). The latter two structures form the core regions of the ventral attentional network (VAN); it could be argued that these two effects are related to each other. They may reflect more automatic control processes initiated primarily by the left PFC, compared to the modulatory influences from the right DLPFC described above. This supports the idea that emotional reactivity may dependent both on the ventral and the dorsal networks (Viviani 2013). The proposed role of the RTPJ as one of the interfaces between both attentional networks (Serences et al. 2005) further supports this claim.

Interestingly, most of the effects regarding reappraisal (REAP minus NEG contrast) were visible only when the efficiency of emotional control was included as a covariate (Fig. 1, flows depicted in black). This shows that much of the unexplained variance could be related to within-group differences in the ability of the participants to control their negative emotions. It also shows that the ability to control emotions, like other cognitive abilities, is highly diversified across population. It would be interesting for future studies to determine which effective connectivity patterns are associated with more effective reappraising.

The second contrast (“passively watching negative clips” minus “watching neutral clips”) showed strengthened links between the dorsal attentional areas (bilateral IPS) and the left DLPFC (Fig. 1, graph “c”). It is possible that this pattern may be a part of automatic control processes which adjust the depth of information processing and facilitate the processing of negative content of high arousal. As suggested by other data, the left DLPFC region may play a dominant role in the continuous monitoring of demands of the current situation and imposing automatic control over attentional systems (Vanderhasselt et al. 2009). Importantly, such control processes may be initiated spontaneously, without awareness, by negative stimuli themselves (Gyurak et al. 2011). Using effective connectivity, we previously found the neural signatures of top-down control processes to be intensified in a state of emotional tension. They involved increased information flow from the left dorsolateral PFC to the attentional and sensory areas when subjects were asked to passively view negatively-valenced IAPS pictures (Wyczesany et al. 2014a). The effect, limited to the left hemisphere, was interpreted as automatic control over attentional system. As the flows in the present study are in the opposite direction, we would argue that the effect is related to the second proposed role of the left DLPFC—monitoring of demands of the current situation. It is possible that in this study the explicit instructions to refrain from using any control strategies resulted in blocking spontaneous control processes. It would be very interesting in future studies to test such automatic processes in a more systematic way, for example by evoking non-conscious goals of controlling emotions. Alternatively, the changes in connectivity seen for this contrast could also reflect automatic responses to negative stimulation, which include appraising its meaning and some of the processes related to generating negative emotions.

In summary, reappraisal was related to increased activity of both left and right DLPFC as sources of neural activations; however, the right hemisphere was more related to influencing DAN (which controls attention voluntary), whereas the left hemisphere to communicating with VAN (which controls attention automatically). This observation, along with other findings, may help elucidate discrepant lateralization patterns obtained in previous studies. Reappraisal may engage the right hemisphere to a greater extend when it is more difficult and more cognitively-demanding to enact. When it is easy to manage, more automatic processes would be involved associated with the left hemisphere. However, when reappraising is more difficult to manage, volitional processes need to be intensified and take over automatic control, heightening the right hemisphere activation. In line with this reasoning, such right-sided activations were found in more demanding conditions when reappraising high- compared to low-intensity pictures (Silvers et al. 2014), decreasing (compared to increasing) emotional responses (Ochsner et al. 2012), or reappraising film clips (rather than pictures; Goldin et al. 2008). Moreover, left DLPFC was also related to automatic emotion processing or controlling processes while passively watching negative film clips, further supporting the role of the left DLPFC in the automatic processes.

Overall, thanks to the novel and advanced EEG method, the study provides a deeper insight into the complex nature of emotional control. To our knowledge, this is the first study using the EEG connectivity method to estimate the effects of voluntary emotional control and one of the few studies using film clips, thought to be more ecologically valid than pictures. However, the study showed some limitations. To maximize emotional effects, our test subjects were women only as they show stronger responses to emotional stimuli (Gardener et al. 2013). As such, the results cannot be easily extrapolated to the male population. We found few if any effects related to the VMPFC or the OFC. These structures are frequently, albeit inconsistently, related to emotional control (Buhle et al. 2013); however, they are difficult (or even impossible) to trace by surface EEG recording. Furthermore, we provided our participants with a free choice in terms of which reappraisal tactic they used. It should be noted that each tactic required slightly different cognitive demands, thus it would be interesting for future studies to consider each of them separately (Shiota and Levenson 2009).

To conclude, the DTF method is shown to be a valuable tool in assessing interactions between control, attentional and perceptual cortical regions. The method revealed that reappraising may be related to both voluntary and automatic control of attention and perceptual regions.
